# ALPPS and simultaneous right hemicolectomy - step one and resection of the primary colon cancer

**DOI:** 10.1186/s12957-015-0544-3

**Published:** 2015-03-27

**Authors:** Mohammad H Fard-Aghaie, Gregor A Stavrou, Kim C Schuetze, Alexandros Papalampros, Marcello Donati, Karl J Oldhafer

**Affiliations:** Department of General and Abdominal Surgery, Asklepios Hospital Barmbek, Ruebenkamp 220, 22291 Hamburg, Germany; Semmelweis University, Asklepios Campus Hamburg, Lohmuehlenstrasse 5, 20099 Hamburg, Germany

**Keywords:** ALPPS, *In situ* split, colorectal carcinoma

## Abstract

**Background:**

Resection of the liver is often limited due to the volume of the parenchyma. To address this problem, several approaches to induce hypertrophy were developed. Recently, the ‘associating liver partition and portal vein ligation for staged hepatectomy’ (ALPPS) procedure was introduced and led to rapid hypertrophy in a short interval. Additionally to the portal vein occlusion, the parenchyma is transected, which disrupts the inter-parenchymal vascular connections.

Since the first description of the ALPPS procedure, various reports around the world were published. In some cases, due to the high morbidity and mortality, a decent oncologic algorithm is not deliverable in a timely manner. If a patient is to be treated with a liver-first approach, the resection of the primary could sometimes be severely protracted. To overcome the problem, a simultaneous resection of the primary tumor and step one of ALPPS were performed.

**Case presentation:**

A 73-year-old male patient underwent portal vein embolization (PVE) after suffering from a synchronous hepatic metastasized carcinoma of the right colic flexure in order to perform a right trisectionectomy. Sufficient hypertrophy could not be obtained by PVE. Thus a ‘Rescue-ALPPS’ was undertaken. During step one of ALPPS, we simultaneously performed a right hemicolectomy. The postoperative course after the first step was uneventful, and sufficient hypertrophy was achieved.

**Conclusion:**

In order to achieve a macroscopic disease-free state and lead the patient as soon as possible to the oncologic path (with, for example, chemotherapy), sometimes a simultaneous resection of the primary with step one of the ALPPS procedure seems justified. A resection of the primary with step two is not advisable, due to the high morbidity and mortality after this step. This case shows that a simultaneous resection is feasible and safe. Whether other locations of the primary should be treated this way must be part of further investigations.

## Background

The first ‘associating liver partition and portal vein ligation for staged hepatectomy’ (ALPPS) was performed in 2007 [1]. Besides the classical portal vein embolization/ligation or two-stage hepatectomy, this new procedure allows rapid liver growth in a short period of time. The portal vein is occluded and the parenchyma transected during step one. This disruption of the inter-parenchymal vasculature is believed to play a major role in the massive hypertrophy in a short interval, enabling a second stage clearing operation in 8 to 14 days. ALPPS has a dramatic impact on surgery for primary and secondary cancers of the liver. Patients, who were once deemed irresectable, are now surgically treatable [2].

Since the original description by Schnitzbauer *et al*., various reports from around the world were published with overwhelming enthusiasm [1,3]. Nevertheless, due to the high morbidity of the procedure, there have also been critical positions [4]. Therefore, ALPPS remains controversial.

After the medium-term outcomes were reported, the euphoric wave abated. Recently, we published our results of ten patients: Seven of these patients were followed up, and six of them showed early local recurrence or generalized disease. We concluded that, although higher resectability rates are achieved, these patients are at high risk for recurrence [5]. However, in some patients, the ALPPS procedure remains the only option for surgical treatment [6].

Patients with synchronous liver metastases have a poor outcome. To overcome the issue of the high tumor burden in the liver, the liver-first approach was suggested. This reversed approach showed some promising results in this highly endangered group [7].

Where do ALPPS and liver-first approach get together? In general, if patients with colorectal cancer should be treated with a liver-first approach, postoperative complications after liver resection can prevent a planned oncologic therapy. The time between resection of the liver metastases and the resection of the primary can protract immensely [8-10]. This problem can be solved with the combination of the first step of ALPPS and removal of the primary tumor [11].

## Case presentation

**Figure 1:**
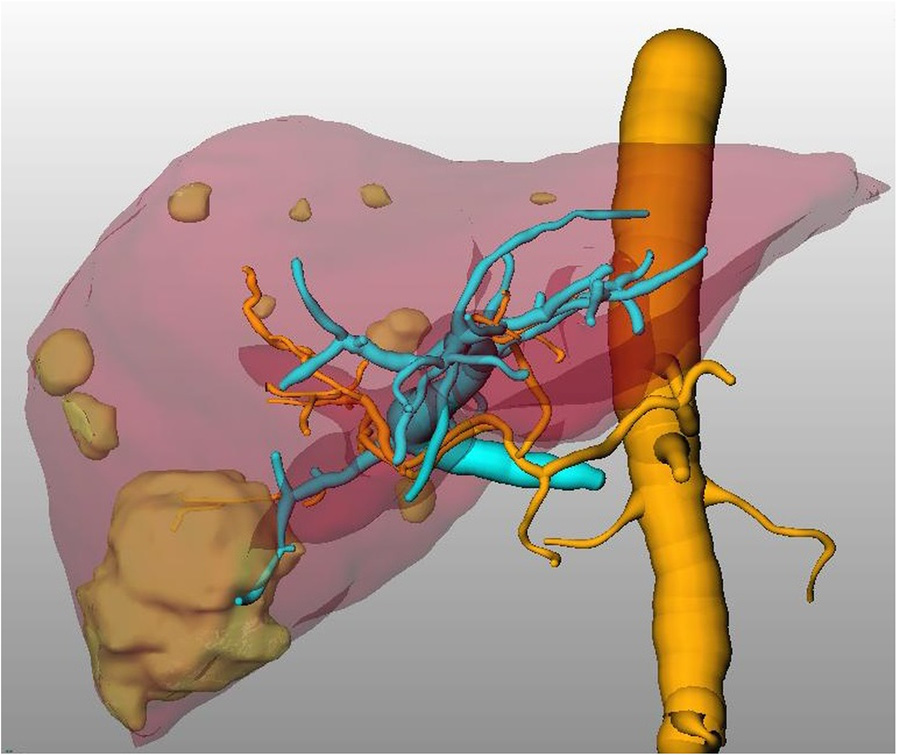
**Three-dimensional reconstructed CT showing the masses with the aorta, hepatic artery, and portal vein.**

An otherwise healthy and asymptomatic 73-year-old patient was referred to our service with a synchronous hepatic metastasized carcinoma of the right colic flexure. The initial staging showed five metastases in the right hemi-liver; the largest metastasis in segment 6 measured 4.5 × 6.5 × 5 cm (staging according the 7th edition of the UICC: cT3, cN1, cM1). In order to achieve hepatic clearance, a right trisectionectomy was necessary (Figure 1).

The total liver volume (TLV) was 1.485 ml, and the volume of the left lateral section, the future liver remnant volume (FLRV), was 189 ml. Thus, sufficient hypertrophy of the left lateral section was necessary.

The multi-disciplinary team (MDT) suggested a liver-first approach consisting of portal vein embolization and neoadjuvant FOLFOX (folinic acid, fluoruracil, oxaliplatin) therapy, then re-staging after 2 months, and if sufficient hypertrophy was obtained, a right trisectionectomy is performed.

The patient received four cycles of neoadjuvant FOLFOX; and 2 weeks after the beginning of the chemotherapy, the PVE was performed.

After 2 months, the FLRV was only 319 ml (20% of TLV) in spite of PVE. Therefore, we decided to achieve sufficient hypertrophy via a Rescue-ALPPS. Four weeks after the last cycle of chemotherapy, the first step of ALPPS plus right hemicolectomy were performed without any perioperative complications.

The operation was started with ALPPS. After identification of the portal structures, the right portal vein was transected. The hepatic artery and common bile duct were marked with loops. The parenchyma was transected at the falciforme ligament and the middle hepatic vein dissected. A Pringle maneuver was not used. After step one, the right hemicolectomy was performed with a side-to-side ileo-transversostomy. The blood loss in step one was 700 ml. The histology of the FLR showed a low-grade periportal fibrosis after chemotherapy.

The patient was discharged after 13 days. CT volumetry on day 8 after step one revealed only 25% FLR, therefore we postponed the second step of ALPPS. The CT 25 days post-op showed a FLRV of 477 ml (31% of TLV). Thus, the second step of ALPPS was performed the following day. There were no intraoperative complications, and the blood loss was 350 ml. The postoperative histology revealed pT4a, pN1b (2/15), cM1, L1, V0, G2, and R0.

**Figure 2:**
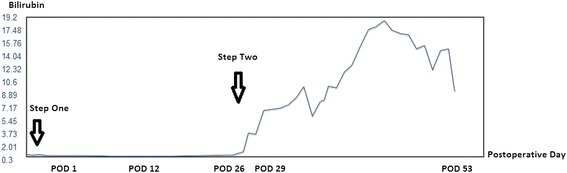
**Bilirubin (mg/dl) curve over the patient’s course.**

The postoperative course after the second step of ALPPS was severely disturbed and ultimately fatal. Initially, the bilirubin was 6.7 mg/dl at the 3rd postoperative day (POD) and climbed to 8.5 mg/dl at 7th POD with no clinical signs of liver failure. Liver function slowly decreased, and bilirubin went up to 15.8 mg/dl at 22nd POD (Figure 2). The international normalized ratio (INR) was 1.5 on 2nd POD, 1.64 on 9th POD, and 1.54 on 15th POD. On 23rd POD, as imaging and endoscopy revealed a possible ischemic bile duct stricture, we explored the abdomen and performed a hepaticojejunostomy. The histology revealed high grade cholestasis. Finally, the patient suffered from acute liver failure, proven by histology, due to a combination of small for size syndrome and cholestasis caused by an ischemic bile duct stricture and died 27 days after step two.

## Conclusions

To our knowledge, there are only few reports of simultaneous step one of ALPPS and primary tumor resection. We discussed the problem of the liver-first approach, especially if combined with ALPPS, and therefore decided to confront it with a simultaneous resection of the primary. Nevertheless, the final decision to perform a resection of the primary with step one was made during surgery, as the first step of ALPPS was straightforward and without complications.

The postoperative course after the first step confirmed the rightfulness of this decision. The patient was even discharged after 13 days.

Can the resection of the primary tumor be done in step two? There is a lack of data to support or refute this approach, but considering our own experience after the second step of ALPPS and published data by Schadde *et al*., a resection of the primary at step two does not seem feasible or justified [12]. In their multicenter study, Schadde *et al*. reported a development of liver failure only after the second step of ALPPS. The postoperative course of our patient after the second step supports this outcome. The regeneration after the second step is, in some cases, severely disturbed and hepatic failure possible [8,12].

Where to draw the line? A proximal anastomosis of the colon has a lower rate of anastomotic failure than an anastomosis of the rectum [13]. Undoubtedly, the trauma of a low anterior resection is far bigger than that of a resection of the colon. Therefore, if the patient had suffered from rectal cancer, a resection of it would not be an option.

The patient developed a hepatic failure after the second step due to a small for size syndrome that was severed by an ischemic bile duct stricture. From our point of view, the simultaneous right hemicolectomy was not the cause for the severe postoperative course. The small for size syndrome shows impressively that the volume of the liver should not be the only factor to determine resectability. Furthermore, the hypertrophy after the portal vein embolization and step one of ALPPS was severely delayed (25 days between step one and step two), suggesting a disturbed synthesis beforehand. Judging from the histology of the FLR during step one, this seems not to be a chemotherapy-related issue.

We believe, if a resection of the primary tumor does not compromise the split procedure, it can be done without any hesitation. Whether other locations of primary tumors can also be operated on simultaneously, must be part of further investigation.

Due to the postoperative course after step two, this case is arguably not a good example for our suggested algorithm. Nevertheless, the postoperative course after step one confirms the idea to resect the primary simultaneously. The case shows that the complexity of this approach requires a dedicated center.

## Consent

Written informed consent was obtained from the patient for publication of this case report and any accompanying images. A copy of the written consent is available for review by the Editor-in-Chief of this journal.
